# Optimization of window settings for coronary arteries assessment using spectral CT-derived virtual monoenergetic imaging

**DOI:** 10.1007/s11547-024-01835-6

**Published:** 2024-06-27

**Authors:** Tommaso D’Angelo, Domenico Mastrodicasa, Ludovica R. M. Lanzafame, Ibrahim Yel, Vitali Koch, Leon D. Gruenewald, Simran P. Sharma, Velio Ascenti, Antonino Micari, Alfredo Blandino, Thomas J. Vogl, Silvio Mazziotti, Ricardo P. J. Budde, Christian Booz

**Affiliations:** 1 Diagnostic and Interventional Radiology Unit, BIOMORF Department, University Hospital “Policlinico G. Martino”, Via Consolare Valeria 1, 98100 Messina, Italy; 2 Department of Radiology and Nuclear Medicine, Erasmus MC, Doctor Molewaterplein 40, 3015 GD Rotterdam, The Netherlands; 3 Department of Radiology, Stanford University School of Medicine, 453 Quarry Rd, MC 5659, Palo Alto, CA 94304-5659 USA; 4 Division of Experimental Imaging, Department of Diagnostic and Interventional Radiology, University Hospital Frankfurt, Theodor-Stern-Kai 7, 60590 Frankfurt, Germany; 5 Department of Radiology, Policlinico Universitario Ospedale Maggiore, Via Francesco Sforza 35, 20122 Milan, Italy

**Keywords:** Dual-Energy X-Ray absorptiometry, Image interpretation, Computer-Assisted, Computed tomography angiography

## Abstract

**Purpose:**

To determine the optimal window setting for virtual monoenergetic images (VMI) reconstructed from dual-layer spectral coronary computed tomography angiography (DE-CCTA) datasets.

**Material and methods:**

50 patients (30 males; mean age 61.1 ± 12.4 years who underwent DE-CCTA from May 2021 to June 2022 for suspected coronary artery disease, were retrospectively included. Image quality assessment was performed on conventional images and VMI reconstructions at 70 and 40 keV. Objective image quality was assessed using contrast-to-noise ratio (CNR). Two independent observers manually identified the best window settings (B-W/L) for VMI 70 and VMI 40 visualization. B-W/L were then normalized with aortic attenuation using linear regression analysis to obtain the optimized W/L (O-W/L) settings. Additionally, subjective image quality was evaluated using a 5-point Likert scale, and vessel diameters were measured to examine any potential impact of different W/L settings.

**Results:**

VMI 40 demonstrated higher CNR values compared to conventional and VMI 70. B-W/L settings identified were 1180/280 HU for VMI 70 and 3290/900 HU for VMI 40. Subsequent linear regression analysis yielded O-W/L settings of 1155/270 HU for VMI 70 and 3230/880 HU for VMI 40. VMI 40 O-W/L received the highest scores for each parameter compared to conventional (all *p* < 0.0027). Using O-W/L settings for VMI 70 and VMI 40 did not result in significant differences in vessel measurements compared to conventional images.

**Conclusion:**

Optimization of VMI requires adjustments in W/L settings. Our results recommend W/L settings of 1155/270 HU for VMI 70 and 3230/880 HU for VMI 40.

## Introduction

Coronary computed tomography angiography has emerged as a valuable non-invasive tool for the diagnosis of coronary artery disease in symptomatic patients with low to intermediate pre-test risk, especially when clinical evaluation and functional testing are inconclusive [1]. In recent years, the use of dual-energy CT (DECT) has witnessed remarkable growth in clinical practice [2]. Among the available DECT scanners, dual-layer CT is one of the more recently developed configurations. Dual-layer CT scanners include a single X-ray source operating at a constant tube voltage alongside a double-layer detector to differentiate between low and high-energy photons, providing valuable insights into tissue composition and unlocking a diverse range of applications [3–5]. The reconstruction of virtual monoenergetic images (VMI) at a selected energy level is one of the most relevant DECT applications. VMI reconstructions closely resemble conventional 120 kilovolt (kV) polyenergetic images [6]. Previous studies have shown that compared to 120 kV images, VMI reconstructed at 70 and 40 kiloelectron volt (keV) significantly increased contrast-to-noise ratio (CNR) and improved the overall image quality [7, 8]. Notably, low-keV VMI reconstructions increase intravascular contrast attenuation, since the energy level is closer to the k-edge of iodine [9–12]. However, the enhanced vascular attenuation associated with low-keV VMI necessitates adjustments in the display window settings to achieve image quality similar to that obtained with conventional settings.

To the best of our knowledge, standardized window/level (W/L) settings for DE-CCTA-derived VMI reconstructions have yet to be determined. We aimed to determine the optimal W/L settings for the evaluation of coronary arteries. In addition, we sought to evaluate how these W/L settings affected image quality compared to conventional datasets and VMI reconstructions displayed using standard window settings.

## Materials and methods

### Patient population

**Fig. 1:**
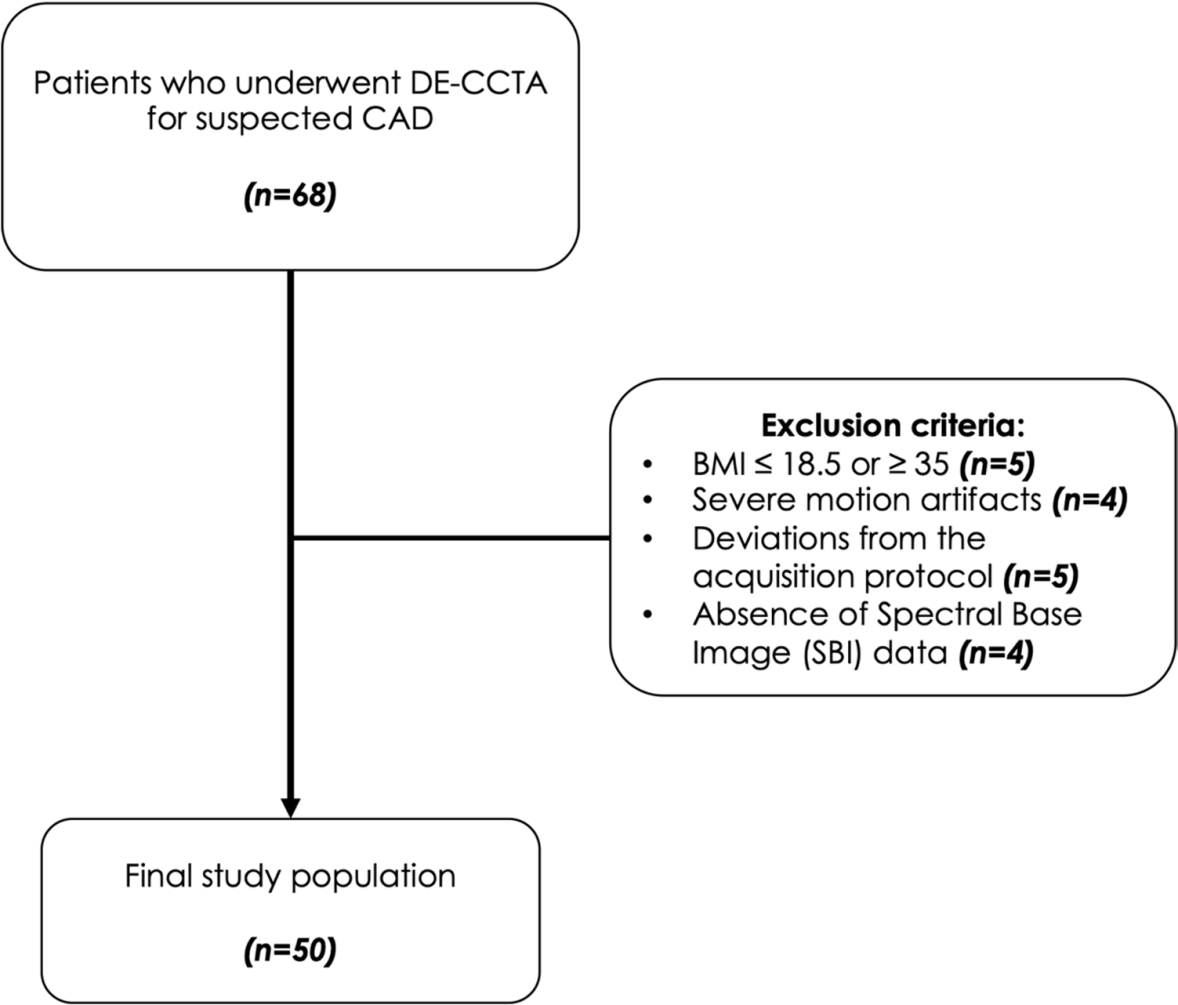
Study flowchart

We included consecutive patients with suspected coronary artery disease who underwent DE-CCTA at our institution between May 2021 and June 2022 (Fig. 1). A waiver for informed consent was obtained for this IRB-approved retrospective study.

### DECT acquisition protocol and patient preparation

CT acquisitions were performed using a 64-row dual-layer CT scanner (IQon CT, Philips Healthcare). Patients were scanned in supine position, in the cranio-caudal direction. Firstly, a prospective ECG-gated unenhanced scan (120 kVp tube voltage; 40 mAs tube current) was performed for the assessment of coronary artery calcium. Subsequently, contrast-enhanced scans were performed after intravenous administration of contrast agent (Iomeprol 400, Iomeron, Bracco) with predefined target iodine delivery rates based on patient’s BMI, as previously described by Mihl et al. [9]. Automated bolus tracking was used, placing a region of interest in the descending aorta using a 110 HU threshold level and a 9 s delay from bolus detection. Tube voltage was set to 120 kVp, mAs was adapted on patients’ body weight, rotation time was 0.27 s, and field of view was 220–250 mm. Patients received nitrates 5 min prior to DE-CCTA scan. Patients with a heart rate ≥ 70 bpm were treated with intravenous infusion of β-blockers.

### DECT image reconstruction

Contrast-enhanced images were reconstructed at 78% of the R-R interval (end-diastolic phase) with fixed acquisition and reconstruction parameters (slice thickness: 0.67 mm; increment: 0.34 mm; FOV: 250 × 250 mm^2^; matrix: 768 × 768; pixel size: 0.33 × 0.33 mm^2^. Spectral base images were obtained using a dedicated software (Spectral Recon, Philips Healthcare). Based on results from prior studies, two VMI datasets were evaluated at 70 and 40 keV together with conventional dataset. All images were generated using the same iterative model reconstruction level (IMR Level 1, Cardiac, Philips Healthcare) [10, 13, 14].

### Objective image quality

Datasets were evaluated with a dedicated software (IntelliSpace Portal, Version 8.0, Philips Healthcare) on a CT workstation. Rounded regions of interest with a fixed area of 2 mm^2^ were placed in the following locations: aortic root, proximal segments coronary arteries, basal region of the interventricular myocardial septum, and subcutaneous fat. Measured attenuation values (HU) and standard deviations (SD) were calculated two times, averaged, and compared for conventional and VMIs. Contrast to noise ratio (CNR) was calculated for each dataset using the following equation [7]:

$$ \left( {{\text{HU}}_{{{\text{vessels}}}} - {\text{HU}}_{{{\text{myo}}}} } \right)/{\text{SD}}_{{{\text{fat}}}} $$

### Window setting

**Fig. 2:**
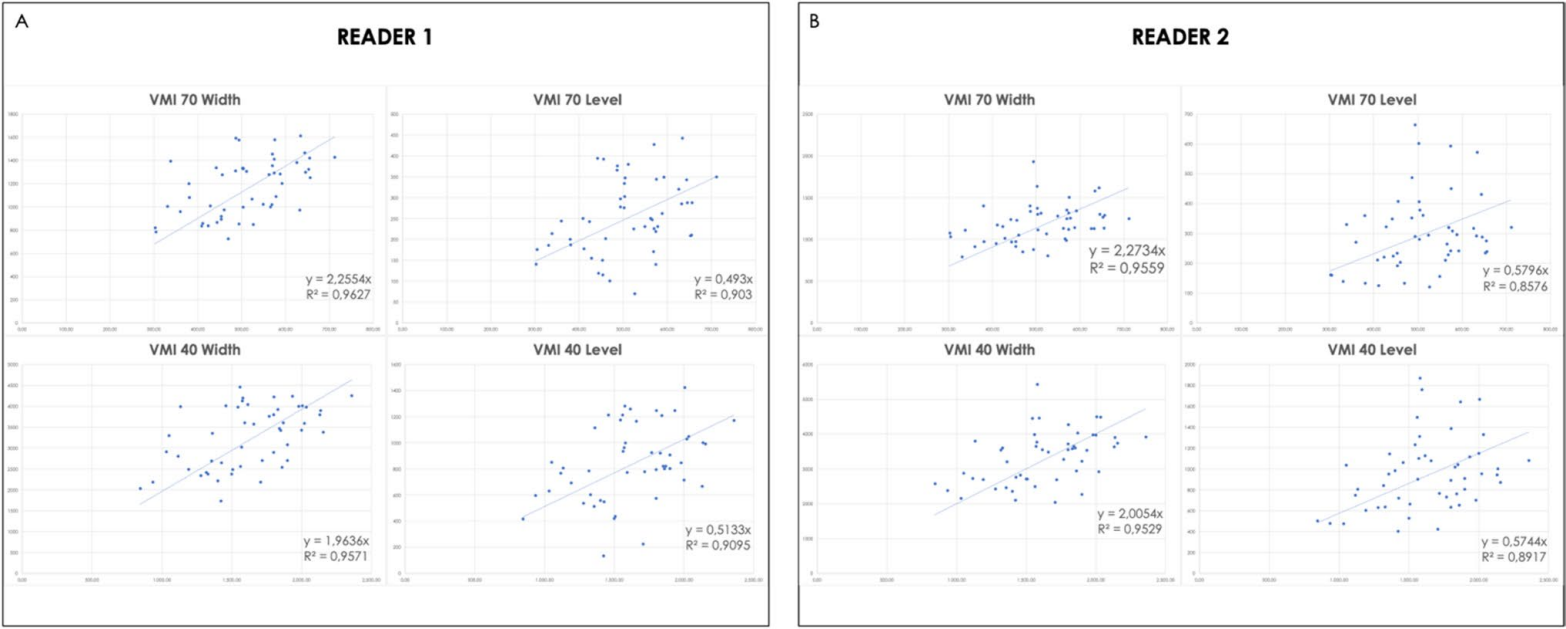
Linear regression analysis scatterplots from reader 1 (**A**) and reader 2 (**B**) derived for both VMI 70 and VMI 40 W/L settings

Given the lack of established optimal windowing values for VMI visualization of coronary vessels, we used conventional images with vendor-suggested windowing values (width: 900 HU; level: 100 HU) as a reference. Two observers with 2 and 7 years of experience in cardiovascular imaging were instructed to manually adjust window settings to achieve the optimal visualization for each VMI reconstruction. Readers were blinded to the energy level of the VMI reconstruction and to examination data. Images were displayed in a dark room, in a random order, and in separate sessions for each reader, with an interval of at least two weeks between conventional and VMI reading to avoid recall bias. Both observers documented their chosen best W/L values for each dataset, which were then averaged to determine the “best W/L” (B–W/L) settings for 70 and 40 keV reconstructions. Secondly, B-W/Ls were normalized to the aortic attenuation values by mean of linear regression analysis [15–17]. Both values were plotted individually for 70 keV and 40 keV VMI datasets. The derived W and L scatterplots were used to produce separate linear regression lines, resulting in first order equations that were used to determine slope values (Fig. 2). These values were then averaged and multiplied by the individual patient’s mean coronary attenuation to obtain “optimized W/L” (O-W/L) values.

### Subjective image quality

Readers assessed subjective image quality of every dataset shown in separate sessions, with the standard window width and level 900 and 100 HU (S-W/L), as recommended by the vendor and O/W-L settings. All observers were blinded to patient data, type of image reconstruction, and window settings to avoid potential bias. Only a single image series per patient was shown during each session. A 5-point Likert scale (1: poor quality, non-diagnostic; 2: sufficient; 3: satisfactory; 4: good; and 5: excellent) was used to evaluate: vascular contrast, stenosis demarcation, blooming, and overall impression. Furthermore, observers were asked to manually measure the maximum diameter of the left main and right coronary arteries on axial CT images for conventional, VMI 70, and VMI 40 datasets, when applying S-W/L and O-W/L, to determine the impact of W/L settings on vessel sizing.

### Statistical analysis

Statistical analysis was performed using a commercially available software (MedCalc Software Ltd, version 20). Data distribution was assessed with the Shapiro–Wilk test. Categorical variables were reported as numbers and percentages, whereas continuous variables were reported as mean ± SD and median and IQRs. Normally distributed data were compared using a two-tailed *t*-test, whereas non-normally distributed data were assessed using the Mann–Whitney *U* test. A *p*-value < 0.05 was used to confirm a statistically significant difference. Repeated measurements of variance were used to evaluate image quality and differences in vessel sizing. Cohen’s kappa coefficient (k) was used to calculate the interobserver agreement between the readers. The differences in measurements were evaluated by means of Bland Altman plots and correlation was assessed by intraclass correlation coefficient (ICC) statistics. ICC results were interpreted as follows: ICC < 0.40 = poor correlation, ICC = 0.41 − 0.59: fair correlation, ICC = 0.60 – 0.74: good correlation, ICC > 0.75 = excellent correlation.

## Results

### Patient population

From an initial cohort of 68 consecutive patients, 18 were excluded according to the following criteria: BMI ≤ 18.5 or ≥ 35 (n = 5); absence of spectral base image data (n = 4); deviations from the DE-CCTA acquisition protocol (n = 5); severe motion artifacts (n = 4). The final study population consisted of 50 patients (30 males; mean age 61.1 ± 12.4 years). Mean body mass index was 25.7 ± 3.6 kg/m^2^. Calcified coronary lesions were present in 33 (50%) patients (Table 1).

**Table 1:** Demographic characteristics of the study population

Characteristics	All	Men	Women
Patients, n (%)	50 (100%)	30 (60%)	20 (40%)
Age (years), Mean (SD)	61.1 (12.4)	61.9 (11.6)	59.9 (13.6)
BMI (kg/m^2^), Mean (SD)	25.7 (3.6)	26.3 (3.8)	24.9 (3.1)
18.5–24.9: n (%)	25 (50%)	14 (28%)	11 (22%)
25–29.9: n (%)	20 (40%)	12 (24%)	8 (16%)
30–34.9: n (%)	5 (10%)	4 (8%)	1 (2%)

*BMI* body mass index, *SD* standard deviation

### Objective image quality

Mean attenuation in the coronary vessels for conventional images (aorta: 444.4 ± 61.7 HU; overall coronary vessels: 463.2 ± 78 HU) was lower compared to VMI 70 (aorta: 515.6 ± 81.5 HU—*p* < 0.0001; overall coronary vessels: 511.1 ± 91.8 HU; *p* = 0.0060) and VMI 40 (aorta: 1.649.9 ± 283.8 HU—*p* < 0.0001; overall coronary vessels: 1.585.4 ± 303.9 HU; *p* < 0.0001). VMI 70 showed the lowest noise (8.8 ± 2.3 HU) compared to conventional and VMI 40 (all *p* < 0.0001). No significant differences for image noise were observed between conventional and VMI 40 (11.8 ± 3.1 versus 11.1 ± 3.4 HU; *p* = 0.2171). Overall coronary artery CNR of VMI 40 (135.6 ± 46.2) was significantly higher in comparison to conventional (34.8 ± 11.6; *p* < 0.0001) and VMI 70 (51.1 ± 14.6; both *p* < 0.0001). Results of the objective image quality analyses are summarized in Table 2

**Table 2:** Objective image quality

	Conventional	VMI 70	VMI 40	Conventional versus VMI 70	Conventional versus VMI 40	VMI 70 versus VMI 40
HU aorta	444.4 ± 61.7	515.6 ± 81.5	1649.9 ± 283.8	*p* < 0.0001*	*p* < 0.0001*	*p* < 0.0001*
HU overall coronary vessels	463.2 ± 78	511.1 ± 91.8	1585.4 ± 303.9	*p* = 0.0060*	*p* < 0.0001*	*p* < 0.0001*
Noise	11.8 ± 3.1	8.8 ± 2.3	11.1 ± 3.4	*p* < 0.0001*	p = 0.2171	*p* < 0.0001*
CNR	34.8 ± 11.6	51.1 ± 14.6	135.6 ± 46.2	*p* < 0.0001*	*p* < 0.0001*	*p* < 0.0001*

*HU* Hounsfield units, *VMI* virtual monoenergetic images, *CMR* contrast-to-noise ratio

Asterisk shows statistically significant difference

### Window settings

For VMI 70, B-W/L values were 1178.4 ± 220.1 HU/277.4 ± 96.9 HU, from which we derived a rounded B-W/L of 1180/280. For VMI 40, B-W/L were 3293.7 ± 699.7 HU/899.1 ± 289.5 HU, resulting in a rounded B-W/L of 3290/900 HU.

Linear regression analysis results were correlated with aortic attenuation resulting in the following equations:

- *Optimized W for* VMI *70: 2.2644* × *HU value*
- *Optimized L for* VMI *70: 0.5363* × *HU value*
- *Optimized W for* VMI *40: 1.9845* × *HU value*
- *Optimized L for* VMI *40: 0.54385* × *HU value*

**Fig. 3:**
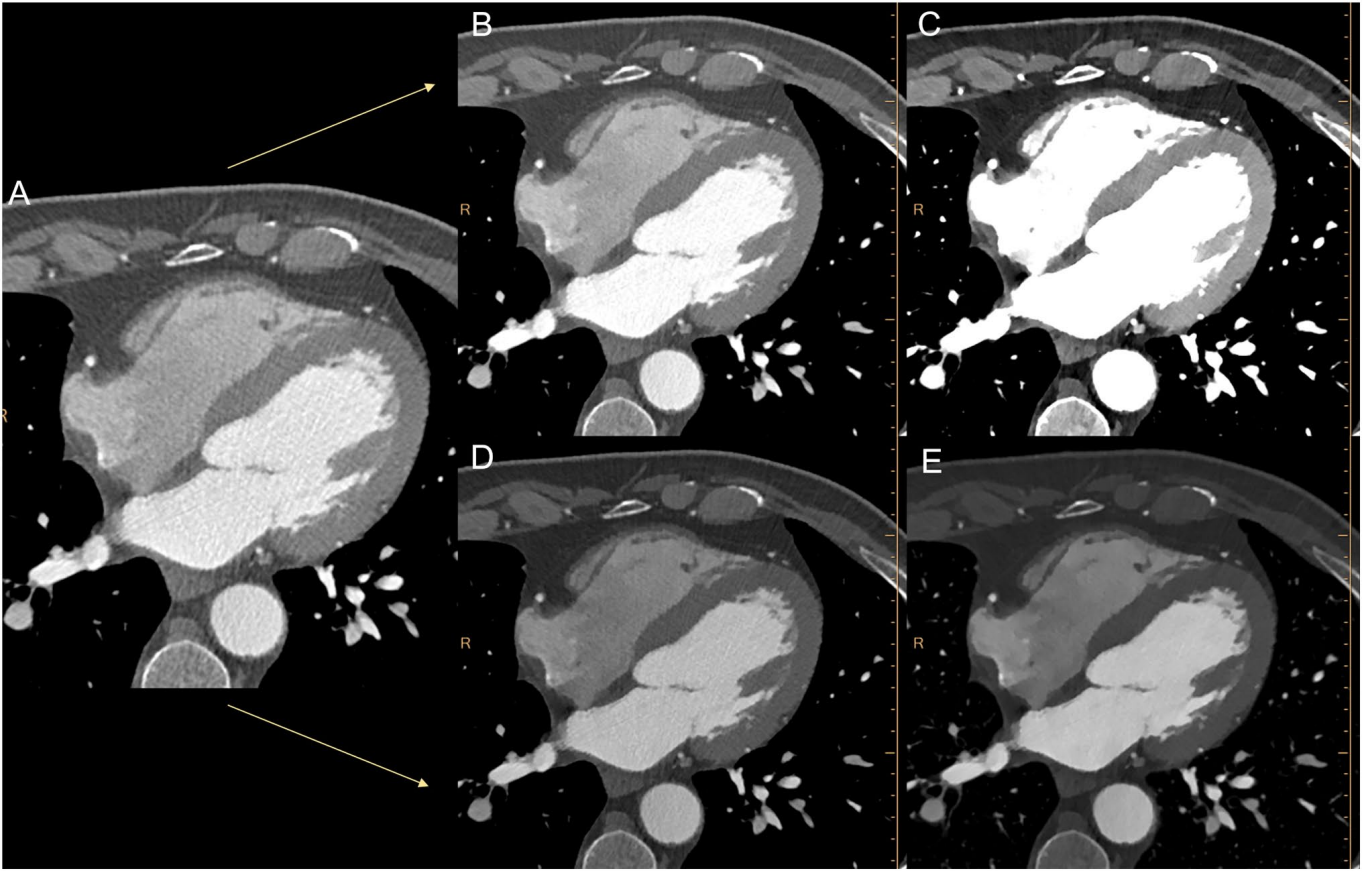
Axial DE-CCTA conventional (**A**) and VMI at 70 (**B**) and 40 (**C**) keV reconstructions displayed with standard window settings and after the application of optimized window settings (**D** and **E**)

Accordingly, O-W/L settings were derived for VMI 70 multiplying the slope value by the individual mean attenuation of coronary vessels (W: 1155.2 ± 227.1 HU; L: 273.6 ± 53.8 HU), resulting in rounded O-W/L of 1155/270 HU. Similarly, O-W/L were also calculated for VMI 40, (W: 3228.3 ± 683.4 HU; L: 884.7 ± 187.3 HU), resulting in rounded O-W/L values of 3230/880 HU (Fig. 3).

Standard W/L settings were significantly different from best and optimized W/L values, both for VMI 40 and VMI 70 (all *p* < 0.0001). Substantial differences were also detected between VMI 70 and VMI 40 for both the B-W/L (*p* < 0.0001) and O-W/L (*p* < 0.0001). On the other hand, no significant differences were found between B-W/L and the O-W/L, both for VMI 70 and VMI 40 (all *p* ≥ 0.4729). Results are summarized in Table 3.

**Table 3:** Window settings

	Mean Width (HU)	Mean Level (HU)	Comparison	Width	Level
			Conv versus VMI 70 B-W/L	< 0.0001*	< 0.0001*
			Conv versus VMI 70 O-W/L	< 0.0001*	< 0.0001*
			Conv versus VMI 40 B-W/L	< 0.0001*	< 0.0001*
Conventional	900	100	Conv versus VMI 40 O-W/L	< 0.0001*	< 0.0001*
VMI 70 B-W/L	1178.4 ± 220	277.4 ± 96.9	VMI 70 B-W/L versus VMI 70 O-W/L	0.4729	0.7713
VMI 70 O-W/L	1155.2 ± 227.1	273.6 ± 53.8	VMI 70 B-W/L versus VMI 40 B-W/L	< 0.0001*	< 0.0001*
VMI 40 B-W/L	3293.7 ± 699.7	899.1 ± 289.5	VMI 40 B-W/L versus VMI 40 O-W/L	0.4954	0.7119
VMI 40 O-W/L	3228.3 ± 683.4	884.7 ± 187.3	VMI 40 O-W/L versus VMI 70 O-W/L	< 0.0001*	< 0.0001*

*HU* Hounsfield units, *VMI* virtual monoenergetic images, *B-W/L* best window/level, *O-W/L* optimized window/level

Asterisk shows statistically significant difference

### Subjective image quality

VMI 70 and 40 scores were significantly higher compared to conventional when applying O-W/L settings (all *p* ≤ 0.0027), and image quality was rated considerably higher for VMI 40 O-W/L compared to VMI 70 O-W/L for vascular contrast, and overall impression (all *p* ≤ 0.0003). VMI 70 O-W/L obtained the best scores for blooming compared both to conventional and VMI 40 O-W/L (all *p* ≤ 0.0347). Stenosis demarcation ratings were significantly improved for both VMI 70 and VMI 40 by the application of O-W/L, without any significant difference between the two VMI energy levels (*p* = 0.0630). Both VMI 40 O-W/L and VMI 70 O-W/L respectively received higher scores compared to the corresponding VMI 40 S-W/L and VMI 70 S-W/L (all *p* < 0.0001). Inter-observer agreement was moderate to good among all evaluations (all k ≥ 0.60, range 0.60–0.82). Results are summarized in Table 4.

**Table 4:** Subjective image quality

	Vascular contrast	Stenosis demarcation	Blooming	Overall impression
Score and Interobserver agreement (k)				
Conventional	3 (3–3.5) k = 0.70 [0.52–0.88]	2.5 (2–3) k = 0.79 [0.65–0.93]	2.75 (2.5–3) k = 0.61 [0.40–0.83]	3 (2.5 -3) k = 0.60 [0.41–0.79]
VMI 70 S-W/L	3 (3–4) k = 0.77 [0.63–0.92]	2.5 (2–3) k = 0.69 [0.49–0.89]	2.5 (2–3) k = 0.69 [0.49–0.89]	3 (2,5–3) k = 0.74 [0.58–0.90]
VMI 70 O-W/L	4 (4–4) k = 0.76 [0.57–0.94]	4 (3–4) k = 0.66 [0.46–0.87]	3 (3–4) k = 0.63 [0.41–0.86]	4 (3.5–4) k = 0.70 [0.50–0.90]
VMI 40 S-W/L	1.75 (1–2) k = 0.63 [0.42–0.84]	1 (1–2) k = 0.81 [0.63–0.99]	1 (1–1) k = 0.65 [0.34–0.97]	1 (1–1.5) k = 0.67 [0.45–0.90]
VMI 40 O-W/L	4.25 (4–5) k = 0.69 [0.51–0.87]	4 (3–4) k = 0.82 [0.68–0.97]	3 (3–3.5) k = 0.77 [0.58–0.95]	4.5 (4–5) k = 0.80 [0.66–0.95]
Statistical comparison				
VMI 70 O-W/L versus Conventional	*p* < 0.0001*	*p* < 0.0001*	*p* < 0.0001*	*p* < 0.0001*
VMI 70 O-W/L versus VMI 70 S-W/L	*p* < 0.0001*	*p* < 0.0001*	*p* < 0.0001*	*p* < 0.0001*
VMI 40 O-W/L versus Conventional	*p* < 0.0001*	*p* < 0.0001*	*p* = 0.0027*	*p* < 0.0001*
VMI 40 O-W/L versus VMI 40 S-W/L	*p* < 0.0001*	*p* < 0.0001*	*p* < 0.0001*	*p* < 0.0001*
VMI 40 O-W/L versus VMI 70 O-W/L	*p* = 0.0003*	*p = 0.0630*	*p* = 0.0347*	*p* < 0.0001*

*VMI* virtual monoenergetic images, *O-W/L* optimized window/level, *S-W/L* standard window/level

The scores are presented as medians and IQRs; asterisk shows statistically significant difference.

### Vessel sizing analysis

**Fig. 4:**
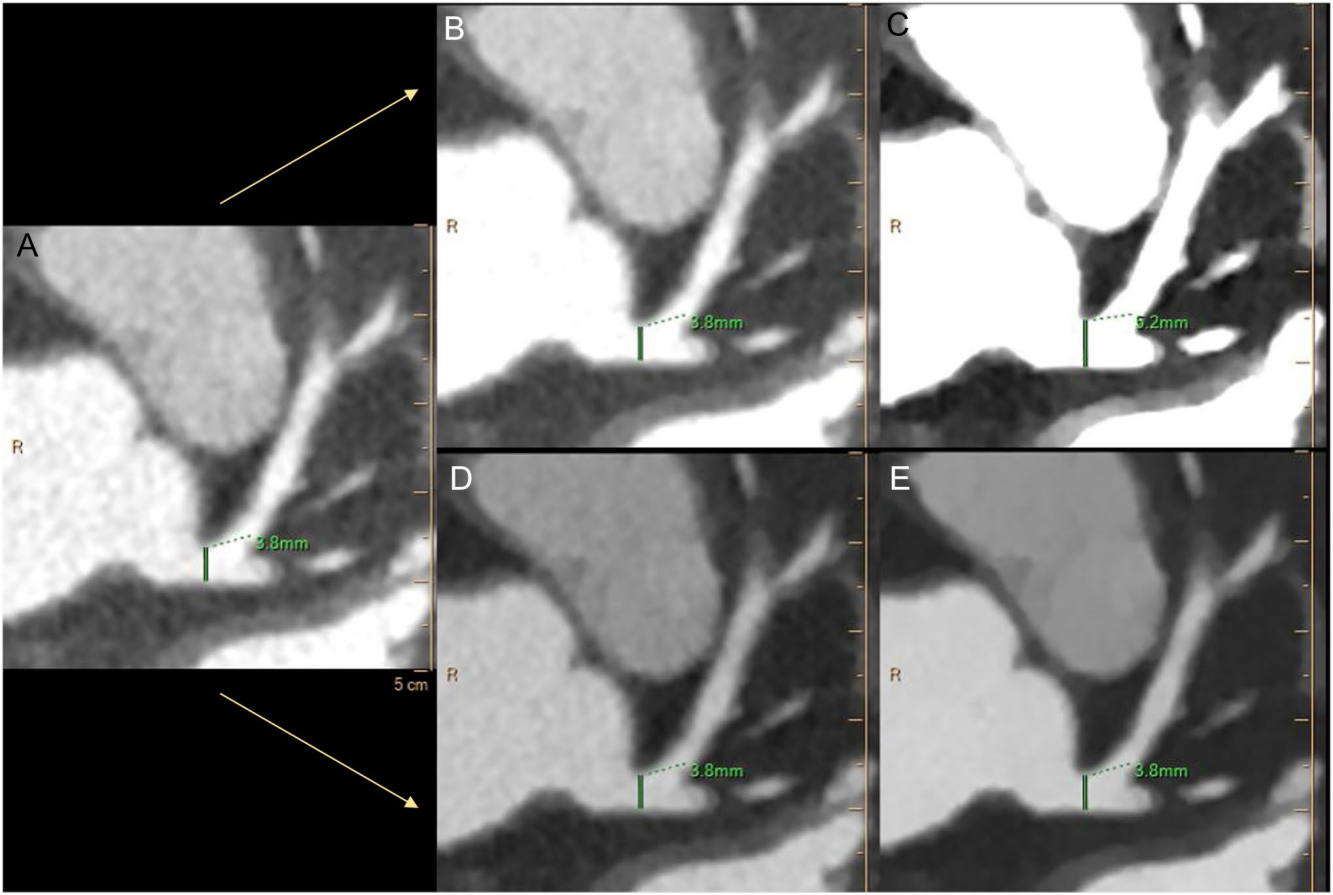
Axial DE-CCTA showing the difference in left main coronary artery diameter (in green) between conventional (**A**) and virtual monoenergetic images at 70 (**B**, **D**) and 40 (**C**, **E**) keV with the application of standard (**B**, **C**) and optimized window settings (**D**, **E**)

A significant overestimation was observed when standard W/L was applied to VMI 40 dataset (all *p* ≤ 0.0003), with a mean difference of 0.63 mm and 0.53 mm respectively for LM and RCA and larger limits of agreement (Table 5). No significant differences in sizing were noted when the O-W/L setting was adopted both for VMI 40 and VMI 70 (all *p* ≥ 0.5116). Figure 4 shows an example of vascular diameter measurement in the three different datasets, before and after the adoption of the O-W/L.

**Table 5:** Coronary vessels diameters

	Conventional	VMI 70 S-W/L	VMI 70 O-W/L	VMI 40 S-W/L	VMI 40 O-W/L
LM (mm)	4.16 ± 0.78	4.18 ± 0.77	4.18 ± 0.80	4.79 ± 0.85	4.21 ± 0.81
RCA (mm)	3.28 ± 0.61	3.31 ± 0.59	3.29 ± 0.63	3.81 ± 0.75	3.35 ± 0.60
Statistical comparison					
	Conventional versus VMI 70 S-W/L	Conventional versus VMI 40 S-W/L	Conventional versus VMI 70 O-W/L	Conventional versus VMI 40 O-W/L	VMI 70 O-W/L versus VMI 40 O-W/L
LM	*p* = 0.8602 ICC = 0.998 [− 0.02 (− 0.11, 0.07)]	*p* < 0.0001* ICC = 0.727 [− 0.63 (− 1.18, − 0.08)]	*p* = 0.9532 ICC = 0.989 [− 0.02 (− 0.24, 0.21)]	*p* = 0.7904 ICC = 0.984 [− 0.05 (− 0.31, 0.22)]	*p* = 0.8063 ICC = 0.996 [− 0.03 (− 0.16, 0.10)]
RCA	*p* = 0.7768 ICC = 0.995 [− 0.02 (− 0.13, 0.09)]	*p* = 0.0003* ICC = 0.682 [− 0.53 (− 1.13, 0.08)]	*p* = 0.8223 ICC = 0.924 [− 0.04 (− 0.51, 0.43)]	*p* = 0.5116 ICC = 0.913 [− 0.07 (− 0.55, 0.41)]	*p* = 0.6658 ICC = 0.982 [− 0.03 (− 0.25, 0.19)]

*LM* left main, *RCA* right coronary artery, *VMI* virtual monoenergetic images, *O-W/L* optimized window/level, *S-W/L* standard window/level

Asterisk shows statistically significant difference

Mean error is reported in square brackets

Limits of agreement are reported in parentheses

## Discussion

The purpose of this study was to identify optimal window settings for displaying VMI reconstructions in CCTA studies. Similarly, to what found in recent literature, our study showed a significant increase of intravascular coronary attenuation using VMI reconstructions at 40 keV, resulting in higher CNR values compared to conventional datasets [2, 10, 13]. Moreover, we demonstrated that optimal visualization of CCTA at lower-keV VMI requires different window setting than conventional datasets [18]. Both VMI 70 and VMI 40 displaying need to increase W and L values compared to conventional images, due to the higher attenuation obtained from lower VMI keV levels the closer we get to the iodine k-edge energy level (33 keV) [19]. To achieve comparable image quality to the standard settings of conventional images, we performed a linear regression analysis to normalize window setting based on the attenuation of the aorta. Optimal W/L values were 1155/270 HU for VMI 70 and 3230/880 HU for VMI 40 O-W/L, respectively.

Similar to the results described by Bae et al. for conventional pulmonary CT angiography, our suggested window W values are approximately twice the attenuation of the aorta, while window L values are about its half [20]. Moreover, our results are also concordant with window values obtained by D’Angelo et al. in their studies on virtual monoenergetic images obtained by dual-source DECT technology in pulmonary and cerebrovascular angiography [16, 21]. The agreement with these studies might demonstrate that these results strictly depend on the VMI energy level adopted rather than the platform used, thus they may potentially be applicable on VMI images acquired with any DECT system as well as the most recent photon-counting CT technology. On the other hand, the adoption of standard window settings to low-keV monoenergetic images was associated with severe blooming, due to the prominent increase in contrast attenuation [22]. This resulted in insufficient image quality for coronary assessment with lower scores at subjective image quality assessment, compared to conventional datasets. However, the adjustment of window settings with optimized values, allowed for higher scores at subjective image quality assessment, both for VMI 70 and VMI 40. In particular, VMI 40 with O-W/L obtained the best rates for intravascular contrast evaluation (4.34 ± 0.62) amongst all datasets, while VMI 70 with O-W/L achieved the best scores for blooming (3.44 ± 0.58). The inter-observer agreement was good for each of the qualitative parameters evaluated. Consequently, virtual monoenergetic reconstructions provide better subjective image quality compared to conventional images when a proper window setting is selected.

On the other hand, improper window W/L settings may be responsible of significant overestimation of coronary vessels diameter. In fact, when standard settings were applied to VMI 40, this led to vessel oversizing or may lead to underestimate a vascular stenosis. However, these differences were not present if optimized window values were applied to VMI datasets [16].

Our study has several limitations. First, our data was derived within the context of a retrospective study design. Second, the number of readers is relatively small. Nonetheless, we attempted to attenuate this limitation by selecting observers with different experience for subjective image quality assessment. Third, our results are based on a single-vendor CT scanner and our acquisition protocol. While dual-layer CT is the latest available DECT platform, our results may need further validation on different DECT systems as well as the most recent photon-counting CT platforms to be applicable; in fact, differences in scanner configuration and spectral separation capability may lead to differences in image quality of VMI [23].

Our study did not aim to assess VMI diagnostic performance for the detection and grading of coronary stenosis. However, our results showed a significant improvement of CCTA image quality using VMI. We believe that a comparative study with coronary angiography is required to determine whether there is any benefit using low-keV images over conventional CT images in clinical routine.

In conclusion, our results showed that VMI reconstructions in DE-CCTA studies require adjustments of window settings for a proper coronary lumen visualization. We recommend O-W/L values of 1155/270 HU for VMI 70 and 3230/880 HU for VMI 40. Additionally, we also suggest the individual application of our W/L slope values for VMI 70 and VMI 40 to adjust coronary vessel contrast attenuation and achieve optimal settings. This adjustment could prove beneficial, especially when coronary attenuation widely differs from that of our sample, as may occur using different contrast injection protocols.

## Abbreviations

B-W/L: Best window/level
CNR: Contrast-to-noise ratio
DECT: Dual energy computed tomography
DE-CCTA: Dual energy coronary computed tomography angiography
HU: Hounsfield unit
O-W/L: Optimized window/level
S-W/L: Standard window/level
SD: Standard deviation
VMI: Virtual monoenergetic images
W/L: Window/level
